# Peritonitis from *Leclercia adecarboxylata*: An emerging pathogen

**DOI:** 10.1002/ccr3.2094

**Published:** 2019-03-18

**Authors:** Sreedhar Adapa, Venu Madhav Konala, Faiza Nawaz, Tariq Javed, Hemant Dhingra, Irene A. Gutierrez, Magda L. Ramirez

**Affiliations:** ^1^ Division of Nephrology The Nephrology Group Fresno California; ^2^ Divison of Medical Oncology, Department of Internal Medicine Ashland Bellefonte Cancer Center Ashland Kentucky; ^3^ Department of Family Medicine Kaweah Delta Medical Center Visalia California; ^4^ Division of Nephrology Kaweah Delta Medical Center Visalia California

**Keywords:** infection, *Leclercia adecarboxylata*, peritonitis

## Abstract

*Leclercia adecarboxylata* can be misidentified as *Escherichia coli*, due to similar biochemical properties. Automated identification systems and mass spectrometry play a very critical role in isolating atypical organisms like *L adecarboxylata*. General guidelines recommend treating *L adecarboxylata *peritonitis for 3 weeks without removal of peritoneal dialysis catheter.

## 1 INTRODUCTION

*Leclercia adecarboxylata* (*L adecarboxylata*) is a gram‐negative bacillus which can rarely cause infections in humans, particularly in immunocompromised individuals. Peritonitis is a very serious complication that is often responsible for catheter loss and switching the dialysis modality in patients undergoing peritoneal dialysis. There has been increased recognition of peritonitis caused by rare organisms. We report a case of 48‐year‐old female patient, who presented with abdominal pain and cloudy effluent. Peritoneal fluid effluent showed elevated white blood cell (WBC) with cell count of 2200 cells/μL (with 96% neutrophils). Peritoneal fluid culture grew *L adecarboxylata*. Patient was treated with intraperitoneal cefazolin for 3 weeks. Posttreatment peritoneal dialysis effluent was clear with WBC count of 2 cells/μL and repeat fluid culture was negative. *L adecarboxylata* is an opportunistic organism, which has been isolated from various body fluids like blood, stool, sputum, urine, peritoneal fluid, and pus. *L adecarboxylata* can be misidentified as *Escherichia coli* (*E coli*), due to similar biochemical properties. Automated identification systems and mass spectrometry in clinical microbiological laboratories play a very critical role in isolating atypical organisms like *L adecarboxylata*, precisely. *L adecarboxylata* is susceptible to most of the common antibiotics; but however, strains producing extended spectrum beta‐lactamases have been reported. *L adecarboxylata* is capable of causing serious and life‐threatening infections. Prompt diagnosis and timely intervention will prevent further complications as in our patient.

*Leclercia adecarboxylata* is a motile gram‐negative bacillus isolated from drinking water, belonging to Enterobacteriaceae family first described in 1962, by Leclerc as Escherichia adecarboxylata.^1^ In 1986, it was renamed *L adecarboxylata* in honor of Leclerc, as it was distinct genus phenotypically and genetically.^2^ *L adecarboxylata* causing infections in humans are very rare, mostly limited to immunosuppressed or with serious chronic medical conditions. This bacterium is susceptible to most of the antibiotics, but cases with antibiotic resistance have been reported. There have been very few cases of *L adecarboxylata* peritonitis reported in the literature.

## 2 CASE REPORT

A 48‐year‐old female with history of end‐stage renal disease secondary to diabetic nephropathy presented with nausea, vomiting, fever, and abdominal pain for two‐day duration. Patient has been on automated peritoneal dialysis for 2 years and never had an episode of peritonitis. Patient lives in a ranch home and takes care of cattle. Other medical problems include the following: hypertension, diabetes, anemia of chronic disease, and coronary artery disease. Patient had low‐grade fever and her other vital signs were stable. Physical examination revealed diffuse abdominal tenderness and no drainage from exit site. No tenderness was elicited along the tunnel of peritoneal dialysis catheter. Peritoneal dialysis effluent showed elevated WBC with cell count of 2200 cells/μL (with 96% neutrophils). Gram stain revealed >100 WBC, and no organisms seen. Patient received empirical treatment with intraperitoneal Vancomycin and Ceftazidime. Effluent grew gram‐negative bacilli, which was identified as *L adecarboxylata* by VITEK mass spectrometry using Matrix Assisted Laser Desorption Ionization Time‐of‐Flight (MALDI‐TOF) technology. The organism was reported to be pan sensitive to antibiotics. Intraperitoneal antibiotic therapy was narrowed to Cefazolin, which was continued for 3 weeks. Posttreatment peritoneal dialysis effluent was clear, with WBC count of 2 cells/μL and repeat fluid culture was negative.

## 3 DISCUSSION

*Leclercia adecarboxylata* is an ubiquitous motile facultative anaerobic gram‐negative bacillus of Enterobacteriaceae family. The organism has been isolated from multiple environmental sources including water, animals, and humans. In our patient, possible source of infection can be from water source or farm animals (cattle,^3^ hen's eggs,^4^ normal gut flora in pigs^5^), which has been reported in literature before.

*Leclercia adecarboxylata* is an opportunistic organism, which can affect patients who are immunocompromised or with chronic medical conditions and have been isolated from various body fluids like blood, stool, sputum, urine, peritoneal fluid and pus.^6^, ^7^ It can present as an isolated organism or as a part of poly microbial infection.^6^ Rarely, it has been reported to cause infections in immunocompetent patients.^6^ *L adecarboxylata* was isolated from blood cultures of an asymptomatic platelet donor.^8^

*Leclercia adecarboxylata* causing peritonitis has been described in the literature, where the patient was treated with intraperitoneal antibiotics, without removal of peritoneal dialysis catheter.^9^, ^10^ It has been reported in patients undergoing hemodialysis with tunneled catheter, which highlights the prevalence of bacteria in patients with end stage renal disease.^11^ It is also known to infect temporary catheters in immunocompromised patients. *L adecarboxylata* is described to cause bacteremia, endocarditis, cholecystitis, and wound infections related to trauma and burns as well as cellulitis.^12^

*Leclercia adecarboxylata* can be misinterpreted as *E coli*, due to similar properties biochemically.^6^, ^7^ It is very important to accurately identify *L adecarboxylata* in the clinical specimen to guide diagnosis, treatment, and epidemiological information.^13^ The epidemiological prevalence of *L adecarboxylata* is under reported until recently, given bacterial assays were not sensitive in differentiating it from *E coli*. Automated identification systems and mass spectrometry in clinical microbiological laboratories play a very critical role in isolating atypical organisms like *L adecarboxylata*, promptly and precisely.^14^ Chromogenic CPSE (translucent agar) media is utilized to differentiate* E coli* from *L adecarboxylata* in small laboratories, where automated expensive laboratory techniques are not available.^14^ Gene sequencing techniques like 16S RNA (Ribonucleic acid) has been used sparsely to confirm the organism.^13^ MALDI‐TOF mass spectrometry helps to identify species better than conventional biochemical systems (92.2% vs 83.1%) and also resulted in better genus identification. MALDI‐TOF mass spectrometry identified 97.7% of Enterobacteriaceae species, precisely.^15^

*Leclercia adecarboxylata* is susceptible to most of the common antibiotics; however, strains producing extended‐spectrum beta‐lactamases have been reported.^16^

As per De Mauri et al, strategies to treat catheter‐related bacteremia are as follows.^11^

**Table:** 

Temporary catheters which can be easily substituted	Short‐course antibiotic monotherapy (1‐2 wk) and replace the catheter
Temporary catheters in complicated patients with a high risk of an adverse result following catheter replacement	Long‐term therapy with two antibiotics and maintain the catheter in situ with constant monitoring
Permanent tunnelled hemodialysis catheters	Long course (2‐4 wk) with at least two intravenous antibiotics, combined with “locked‐in” therapy; retaining the catheter with constant monitoring

## 4 CONCLUSION

*Leclercia adecarboxylata* is a rare cosmopolitan organism, but capable of causing serious and life‐threatening infections. Microbiological techniques have been successful in identifying this organism more frequently. Prompt diagnosis and timely intervention will prevent further complications as in our patient.

## ETHICAL APPROVAL

This is a case report, so exemption has been provided.

## CONFLICT OF INTEREST

The authors declare no conflict of interests.

Informed Consent: Both informed consent and written consent were obtained from the patient.

## AUTHOR CONTRIBUTION

SA, VMK, HD: prepared, reviewed and submitted the Manuscript, TJ, FN, IG, MR: involved in management and preparing case report.
